# A case report of anti-AMPAR encephalitis with involuntary limb shaking and aphasia

**DOI:** 10.1097/MD.0000000000042036

**Published:** 2025-04-04

**Authors:** Shuang Li, Tiantian Huo, Minxia Geng, Jiahao Xing, Tianjun Wang

**Affiliations:** a Graduate School of Hebei North University, Zhangjiakou, Hebei, China; b Department of Neurology, Hebei General Hospital, Shijiazhuang, Hebei, China; c Graduate School of North China University of Science and Technology, Tangshan, Hebei, China; d Graduate School of Hebei Medical University, Shijiazhuang, Hebei, China.

**Keywords:** anti-AMPAR encephalitis, aphasia, immunotherapy, involuntary limb shaking

## Abstract

**Rationale:**

Anti-alpha-amino-3-hydroxy-5-methyl-4-isozolipropionic acid receptor (AMPAR) encephalitis is a rare autoimmune encephalitis mediated by anti-AMPAR antibodies produced on the surface of neuronal cells. Anti-AMPAR encephalitis has a variety of clinical manifestations, the common clinical manifestations are characterized by borderline encephalitis, including short-term memory loss, confusion, behavioral abnormalities, and seizures.

**Patient concerns:**

Here we report a case of anti-AMPAR encephalitis in an adolescent female with aphasia as the first symptom of involuntary limb shaking.

**Diagnoses:**

The diagnosis of anti-AMPAR encephalitis is based on clinical symptoms and further detection of serum antibodies.

**Interventions and outcomes:**

The patient responded well to intravenous methylprednisolone combined with immunoglobulin treatment, and her limbs no longer shook and her speech was fluent.

**Lessons:**

Early diagnosis of autoimmune encephalitis is challenging due to the heterogeneity of clinical manifestations. Our patient was initially diagnosed with involuntary limb movement, so we should conduct further research on symptoms associated with autoimmune encephalitis.

## 1. Introduction

Anti-AMPAR encephalitis is a rare type of autoimmune encephalitis, mainly characterized by borderline encephalitis, including short-term memory loss, confusion, behavioral abnormalities, and seizures. It was first reported in 2009 by Lai^[1]^ et al, but since then there have been few reports at home and abroad, among which involuntary limb movement and aphasia are even rarer. This paper reported a case of an adolescent female patient with involuntary limb shaking as the first symptom accompanied by aphasia, so as to improve neurologists’ understanding of this disease.

## 2. Case report

**Figure 1.**
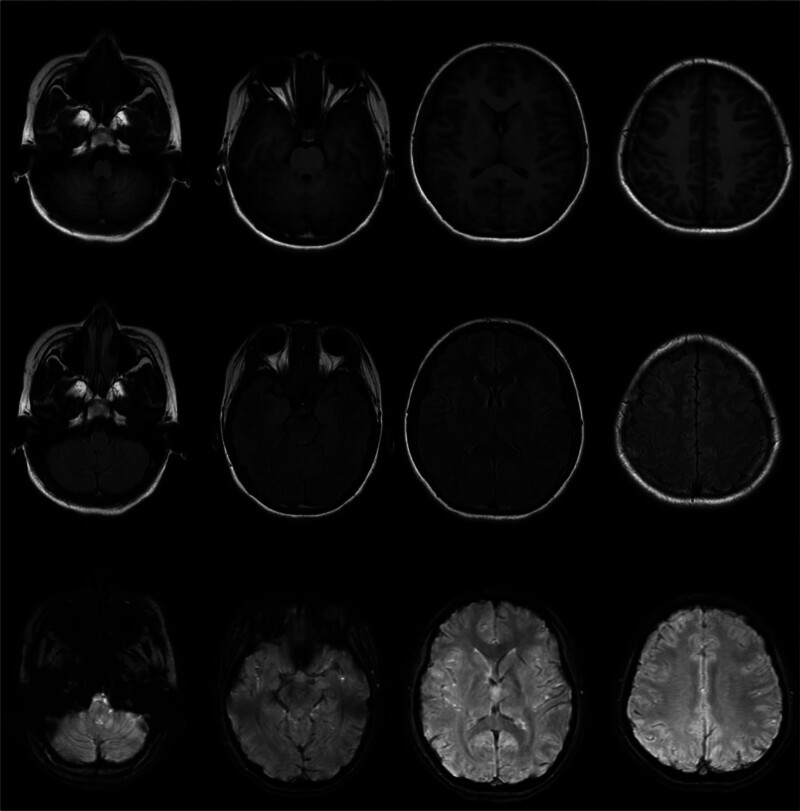
Magnetic resonance imaging (MRI).

**Figure 2.**
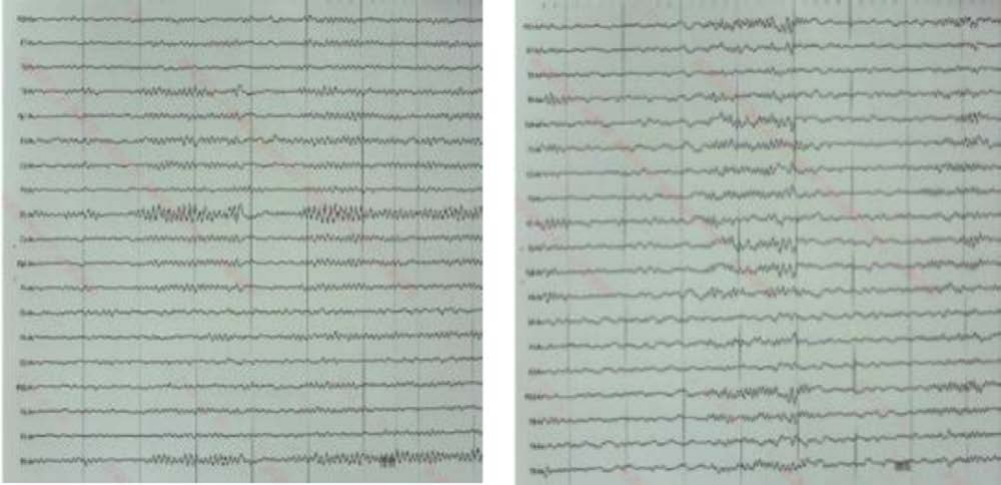
Electroencephalography (EEG).

**Figure 3.**
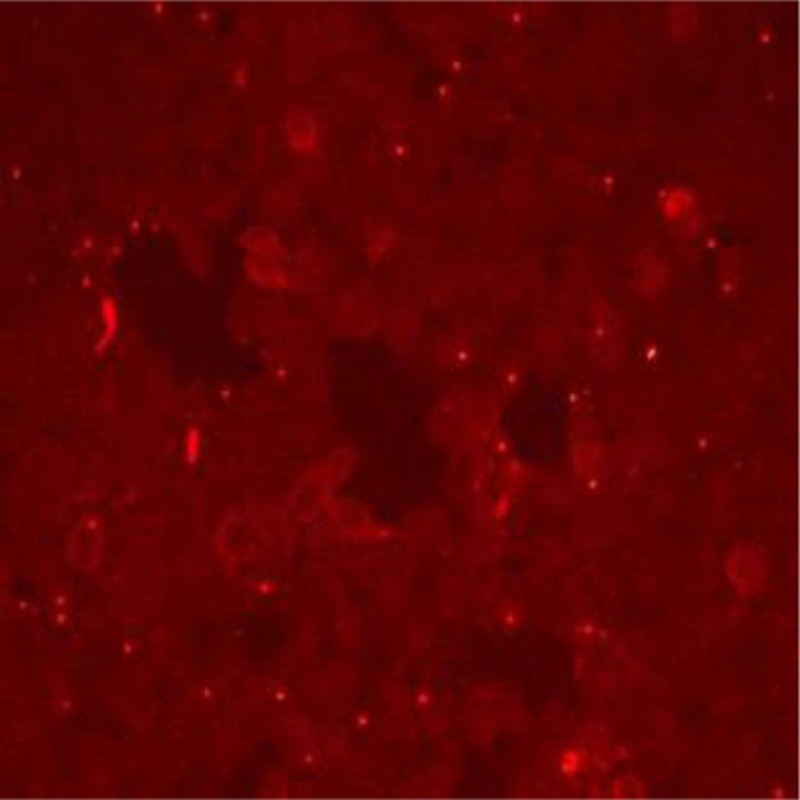
Results of detecting serum anti-AMPAR antibodies by the cell-based assay (CBA) transfection cell method. Judge the level of antibodies through the fluorescence intensity.

**Figure 4.**
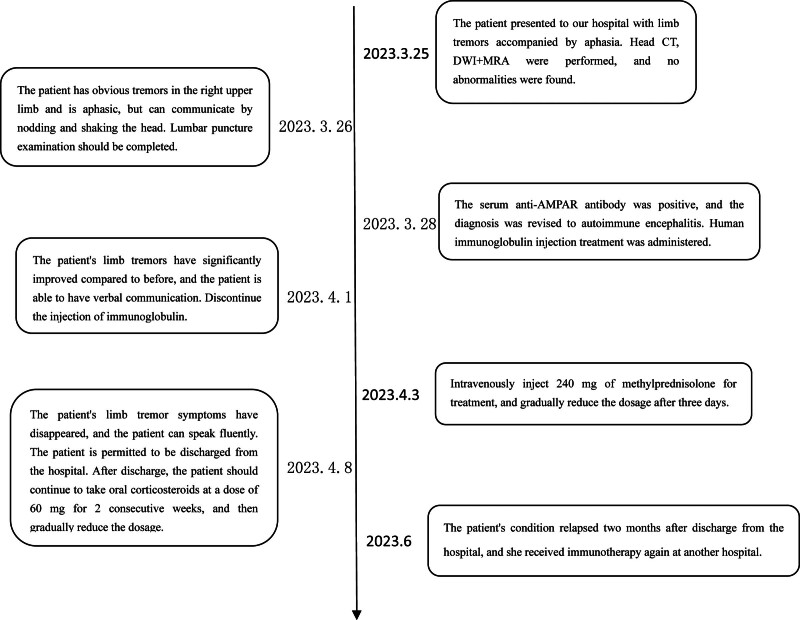
A timeline of medical history.

A 17-year-old female patient was admitted to the neurology department of our hospital on March 25, 2024, mainly due to involuntary limb shaking for 7.5 hours. A 7.5 hours before admission, the patient had involuntary limb shaking without obvious inducement, mainly in the right upper limb. Three hours before admission, the patient had no language, eyes were straight, and symptoms continued to be unsolved. The patient was admitted to the emergency department of our hospital, and no abnormalities were found in skull computed tomography (CT) and skull diffusion-weighted imaging + magnetic resonance angiography. Filed under “Involuntary Movement pending Investigation.” The patient was previously healthy and had a history of upper respiratory tract infection. When admitted to hospital, the body temperature was 37.7 °C, the nervous system physical examination: clear consciousness, silent, lack of cooperation in physical examination, increased muscle tension of limbs, involuntary shaking of both upper limbs, bilateral pathological signs were negative. The initial diagnosis was involuntary limb movement. After admission, ceftriaxone was given anti-infection treatment and ganciclovir antiviral treatment. Complete examination and examination on admission: electrocardiogram: 1. sinus tachycardia 2. generally normal electrocardiogram: urine analysis: ketone body: 1+, abnormal; blood gas analysis, biochemical whole item + SAA + CRP, blood homocysteine, preoperative 8 items, 6 items of thyroid function, serum folic acid, vitamin B12 were not significantly abnormal. Head magnetic resonance imaging (MRI) showed no significant abnormalities (Fig. 1). No significant abnormality was found on cervical vascular ultrasound and subclavian artery ultrasound. On the second day of admission, the head enhanced MRI, electroencephalogram, and lumbar puncture were completed, and the cerebrospinal fluid was examined for autoimmune antibodies and infection-related tests. There were no significant abnormalities in SPGR + CUBE enhanced skull magnetic resonance. There were no obvious abnormalities in the electroencephalogram (Fig. 2). Cerebrospinal fluid biochemistry: cerebrospinal fluid sugar: 79.82 mg/dL↑; there were no obvious abnormalities in the cytology of cerebral spinal fluid (CSF) abscission, CSF antacid ink staining, and CSF analysis. No pathogenic microorganisms were detected by high-throughput sequencing of cerebrospinal fluid. Fecal analysis and occult blood test showed no significant abnormalities. Reexamination urine analysis: leukocyte 2+. On the fourth day of admission, the results of cerebrospinal fluid testing reported 15 serum autoimmune-associated encephalitis antibodies: anti-AMPAR2 antibody 1:10, weakly positive (Fig. 3), cerebrospinal fluid negative. A revised diagnosis was given: autoimmune encephalitis. Methylprednisolone 1 g/day and propyl bulb 20 g/day were given. On the 6th day of admission, the jitter of the patient’s right upper limb was reduced, and the 3 rheumatic factors were improved: antinuclear antibody: 1:100 abnormality: immunoglobulin G: 6.94 g/L↓; blood analysis: white blood cell count 15.67 × 10^9^/L↑, no significant abnormalities were found in all emergency items, no. 1 vasculitis screening, and all female tumors. The patient complained of cough, sputum and elevated white blood cells. Considering the presence of upper respiratory tract infection, ceftriaxone was continued to fight infection. On the 7th day of admission, methylprednisolone was adjusted to 0.5 g/day, and immunoglobulin 20 g/day impact therapy was continued. On the 8th day of admission, a complete chest CT revealed microscopic nodules in the posterior upper lobe and posterior basal lobe of the right lung. Anteroposterior X-ray of pelvis showed no abnormality in pelvic bone structure. Breast ultrasound: there was no obvious space-occupying change in the glandular layers of both mammary glands, and no obvious enlargement of lymph nodes in the armpits of both sides. Gynecologic ultrasound: uterus normal size, polycystic changes in both ovaries. On the 9th day of admission, after 5 days of treatment with methylprednisolone combined with immunoglobulin, the patient’s right upper limb involuntary jittering was reduced, muscle tone was reduced, questions and answers could be answered fluently, and immunoglobulin therapy was discontinued. On the 10th day of admission, methylprednisolone was adjusted to 240 mg/day. There were no abnormalities in the 6 items of perfect hormone. On the 13th day of admission, methylprednisolone was adjusted to 120 mg/day. On the 15th day of admission, the involuntary shaking of the limbs was significantly improved, the muscle strength of the limbs was grade 5, the muscle tension of the limbs was normal, and the speech was fluent. The patient agreed to be discharged from the hospital. After discharge, he was given oral prednisone acetate tablet 60 mg/day. During the recent follow-up, the patient recurred 2 months after discharge and was given immunotherapy again in another hospital.The patient’s diagnosis and treatment timeline is shown in Figure 4.

## 3. Discussion

Anti-AMPAR encephalitis, a subtype of rare autoimmune encephalitis mediated by AMPAR antibodies, was first identified in 2009.^[1]^ Since then, <100 cases have been reported at home and abroad, and the current incidence is unclear. Located in the postsynaptic membrane, AMPAR is an ionic glutamate receptor that plays an important role in plasticity, memory, and learning. AMPAR is mainly composed of 4 subunits (G1uA1–4) and is universally expressed throughout the nervous system, especially in the hippocampus and its limbic regions.^[2]^ AMPAR antibodies act on the cell surface antigens of neurons, namely extracellular antigen epitopes of the receptor GluA1 and GluA2 subunits, selectively reducing the surface synaptic AMPAR cluster and destroying the balance between AMPAR internalization and reinsertion, promoting AMPAR internalization and rapid degradation. AMPAR-mediated synaptic transmission is reduced, which further leads to reduced compensatory and increased endoexcitability of inhibitory synaptic transmission. Antibody induced synaptic and neuronal changes may lead to short-term memory loss and seizures in patients with anti-AMPAR encephalitis.^[3]^ Studies have found that most patients have acute or subacute attacks, which can occur at any age, and the incidence is higher in women than men, most commonly in middle-aged women,^[4]^ and rare in adolescents and children. Only a small number of patients reported a history of preinfection. The clinical manifestations of anti-AMPAR encephalitis vary, with most patients presenting with borderline encephalitis symptoms, including short-term memory loss, confusion, behavioral abnormalities, and seizures. In some patients, dyskinesia can be manifested as gait disorder or ataxia, Parkinson syndrome, and involuntary movement. A few patients have speech disorders, mainly manifested as aphasia. Other manifestations such as insomnia, autonomic dysfunction and dysarthria may also occur,^[5]^ and the underlying mechanism of influence on AMDAR function is not fully understood. Our patient, an adolescent female, presented with involuntary limb shaking as the first symptom, followed by aphasia. AMPAR antibodies are often associated with tumors, and studies have found that 60% of patients have a history or detected tumors, of which thymus tumors are the most common, followed by lung, breast, and ovarian cancer.^[6]^ There are also cases of medullary thyroid carcinoma, melanoma, Ewing sarcoma, spermatogonia, and so on. Tumors are often found after the onset of encephalitis, and tumors are thought to be the trigger for AE, but the mechanism is unclear. Early treatment of tumors is important for good prognosis. No tumor was found during our patient’s treatment, and regular tumor screening is recommended. Brain MRI, electroencephalogram (EEG), CSF examination, and antibody detection are the main diagnostic tools for anti-AMPAR encephalitis.^[7]^ Anti-AMPAR encephalitis magnetic resonance imaging (MRI) is often the most typical manifestation of bilateral temporal lobe T2/fluid attenuated inversion recovery hyperactivity, which may also involve the basal ganglia.^[8]^ The EEG showed focal eclamptic activity in 1 or both temporal lobes, and slow wave rhythm with diffuse or multifocal manifestations, as well as sharp and slow wave abnormalities. Studies have shown that brain MRI and EEG of patients with anti-AMPAR encephalitis may also be completely normal.^[5]^ Imaging and electroencephalogram abnormalities lack specificity for the diagnosis of anti-AMPAR encephalitis. Detection of AMPAR antibodies in blood and cerebrospinal fluid is a clear diagnostic marker for anti-AMPAR encephalitis, and antibodies targeting GluA1 and GluA2 subunits can be detected simultaneously or separately, with GluA2-specific antibodies being more common. The cerebrospinal fluid and imaging findings of some patients were normal.^[9]^ No significant abnormalities were found in brain MRI, EEG, or CSF in our patients, and only AMPAR2 antibody was detected in the blood. At present, there are no reports on the brain histopathology of anti-AMPAR encephalitis. A few cases have reported the presence of GluA1/2 subunits in tumor tissue from patients, and accompanying tumor pathology has been found, which is specifically related to the patient’s antibodies. This suggests that certain types of tumors may play a role in triggering this autoimmune disease. Patients with definite autoimmune encephalitis are recommended to use chest, abdominal, and pelvic CT for cancer screening.^[10]^ In addition, a common feature of anti-AMPAR encephalitis is a tendency to relapse.^[1]^ Our patient recurred 2 months after discharge and was given immunotherapy again. At present, there is no specific treatment plan for anti-AMPAR encephalitis, and other autoimmune encephalitis treatments are usually selected based on reference.^[11]^ Immunotherapy consists of first-line treatment including IVIG, glucocorticoids and plasma exchange, and second-line treatment including rituximab and immunosuppressants. For patients with combined tumors, surgical treatment is the first line of treatment. Patients with poor or recurrent first-line immunotherapy are recommended to receive second-line therapy.^[12]^

Here we report a case of an adolescent female patient with involuntary limb movement as the first symptom accompanied by aphasia. The patient’s blood AMPAR2 antibody was positive, and cerebrospinal fluid, imaging and electroencephalogram were not abnormal. The patient’s tumor was not found during treatment and responded well to hormone combined immunoglobulin therapy, but recurred 2 months after discharge. It is recommended that patients undergo regular tumor screening. Due to the lack of relevant reports on anti-AMPAR encephalitis and the heterogeneity of its clinical manifestations, our understanding of anti-AMPAR encephalitis is limited, and its early diagnosis faces great challenges. Early diagnosis and targeted therapy can help patients get a good prognosis. Here we summarize the clinical manifestations, auxiliary examination and treatment of anti-AMPAR encephalitis in order to improve neurologists’ understanding. In patients suspected of anti-AMPAR encephalitis, lumbar puncture should be performed as soon as possible, and cerebrospinal fluid and serum antibodies should be examined for further confirmation. Immunotherapy and tumor screening are given as soon as possible after diagnosis.

## Acknowledgments

The authors would like to thank Tianjun-Wang for her assistance in writing this manuscript.

## Author contributions

**Conceptualization:** Shuang Li, Tiantian Huo.

**Formal analysis:** Jiahao Xing.

**Project administration:** Minxia Geng.

**Writing – original draft:** Shuang Li.

**Writing – review & editing:** Tianjun Wang.
